# Optimizing Social-Emotional-Communication Development in Infants of Mothers With Depression: Protocol for a Randomized Controlled Trial of a Mobile Intervention Targeting Depression and Responsive Parenting

**DOI:** 10.2196/31072

**Published:** 2021-08-18

**Authors:** Kathleen M Baggett, Betsy Davis, Lisa Sheeber, Katy Miller, Craig Leve, Elizabeth A Mosley, Susan H Landry, Edward G Feil

**Affiliations:** 1 Georgia State University Atlanta, GA United States; 2 Oregon Research Institute Eugene, OR United States; 3 University of Texas Health Sciences Center Houston, TX United States

**Keywords:** maternal depression, parenting, infant social-emotional and social-communication development, mobile intervention, remote coaching, trial protocol, mobile phone

## Abstract

**Background:**

Postpartum depression interferes with maternal engagement in interventions that are effective in improving infant social-emotional and social-communication outcomes. There is an absence of integrated interventions with demonstrated effectiveness in both reducing maternal depression and promoting parent-mediated practices that optimize infant social-emotional and social-communication competencies. Interventions targeting maternal depression are often separate from parent-mediated interventions. To address the life course needs of depressed mothers and their infants, we need brief, accessible, and integrated interventions that target both maternal depression and specific parent practices shown to improve infant social-emotional and social-communication trajectories.

**Objective:**

The aim of this study is to evaluate the efficacy of a mobile internet intervention, Mom and Baby Net, with remote coaching to improve maternal mood and promote parent practices that optimize infant social-emotional and social-communication development.

**Methods:**

This is a two-arm, randomized controlled intent-to-treat trial. Primary outcomes include maternal depression symptoms and observed parent and infant behaviors. Outcomes are measured via direct observational assessments and standardized questionnaires. The sample is being recruited from the urban core of a large southern city in the United States. Study enrollment was initiated in 2017 and concluded in 2020. Participants are biological mothers with elevated depression symptoms, aged 18 years or older, and who have custody of an infant less than 12 months of age. Exclusion criteria at the time of screening include maternal homelessness or shelter residence, inpatient mental health or substance abuse treatment, or maternal or infant treatment of a major mental or physical illness that would hinder meaningful study participation.

**Results:**

The start date of this grant-funded randomized controlled trial (RCT) was September 1, 2016. Data collection is ongoing. Following the institutional review board (IRB)–approved pilot work, the RCT was approved by the IRB on November 17, 2017. Recruitment was initiated immediately following IRB approval. Between February 15, 2018, and March 11, 2021, we successfully recruited a sample of 184 women and their infants into the RCT. The sample is predominantly African American and socioeconomically disadvantaged.

**Conclusions:**

Data collection is scheduled to be concluded in March 2022. We anticipate that relative to the attention control condition, which is focused on education around maternal depression and infant developmental milestones with matching technology and coaching structure, mothers in the Mom and Baby Net intervention will experience greater reductions in depression and gains in sensitive and responsive parent practices and that their infants will demonstrate greater gains in social-emotional and social-communication behavior.

**Trial Registration:**

ClinicalTrials.gov NCT03464630; https://clinicaltrials.gov/ct2/show/NCT03464630

**International Registered Report Identifier (IRRID):**

DERR1-10.2196/31072

## Introduction

Maternal depression during the postpartum period is highly prevalent and is associated with extensive and well-documented effects on parenting behavior and infant developmental outcomes [1-3]. Depressed mothers are more irritable and less responsive to their infants, more likely to make negative attributions for infant crying, show less pleasure in response to infant social bids, and talk less to their infants relative to nondepressed mothers [4-8]. Infants of depressed mothers exhibit more negative and less positive affect, poorer emotion regulation and cognitive development, and less social engagement as well as biological markers associated with subsequent depression [9]. These developmental risks are magnified for the 1 in 6 infants living in poverty, approximately 71% of whom are children of color [10]. In the United States, mothers who are socioeconomically disadvantaged and of nondominant cultures experience depression during the postpartum period at nearly four times the rate of White economically advantaged mothers [11,12], and poverty increases infant susceptibility to the effects of early adverse parenting [13].

Programs designed to foster behavioral and biological foundations of infant and early childhood mental health focus on promoting parent sensitivity and responsiveness [14,15] and have been shown to be effective in improving both parenting behavior and infant developmental outcomes [14-16]. Of particular relevance to this study, the Play and Learning Strategies (PALS) intervention has been shown to increase maternal responsiveness and sensitivity and, thereby, improve infant social-emotional and social-communication behavior and developmental outcomes [17-19]. For infants facing early adversity, intervening early and targeting these nurturing parent behaviors has proven to be effective in promoting infant social-emotional and social-communication trajectories [20-22].

Given these strong outcomes and the associated promise of improving developmental outcomes for at-risk infants, it is of significant concern that the reach of such interventions is low [23,24]. Although home visits are the most common mechanism to support early parenting, particularly for low-income families, we know that these programs reach on average less than 4% of the population in need of such intervention and, in some cases, are prohibitively costly to bring to scale with sufficient intensity [25]. Maternal depression interferes with engagement in early intervention and is effective in improving infant social-emotional and social-communication outcomes [26,27]. Moreover, we know that relatively few depressed mothers access treatment for depression [1,26,28-30], with disparities in the receipt of treatment found for mothers of nondominant cultures and those living in socioeconomic disadvantage [31-34]. Within this context, it is notable that infant parenting interventions and treatment for depression typically operate in silos such that mothers and their infants, who are least resourced and most in need of these interventions, may be least likely to access and engage in them [34].

Although there are effective interventions for depressed mothers that include parenting components [35-38], there is a striking absence of accessible, integrated, and evidence-based interventions that target both perinatal depression and parenting practices that have been demonstrated to optimize infant social-emotional and social-communication trajectories. Moreover, remote service delivery approaches are needed to overcome access barriers that differentially affect women, minorities, and the poor [39-41]. Web-based remote coaching interventions can overcome logistical barriers that often prevent low-income mothers from participating in community-based programs, including lack of transportation and childcare as well as inflexible work schedules [42]. Increased access is particularly possible when interventions are made accessible through smartphones, which diminish or eliminate the digital divide [43].

In our previous programmatic research, we developed a highly effective, guided internet and remote coaching intervention to improve the accessibility of treatments for maternal depression (Mom-Net) [37,44] and promotion of infant and early childhood mental health (e-Play and Learning Strategies [ePALS] BabyNet) [23,45]. To address the existing silo in interventions for maternal depression and early parenting, as well as improve accessibility to these interventions, we created a mobile internet intervention (Mom and Baby Net [MBN]) [46]. MBN integrates our evidence-based guided internet intervention program targeting maternal depression [37,44] with our evidence-based, parent-mediated intervention targeting parent practices that promote infant social-emotional and social-communication competencies [45].

## Methods

### Study Aim and Setting

The primary aim of this study is to evaluate the impact of the MBN intervention on changes in maternal depression, parenting strategies and knowledge, as well as infant social-emotional and social-communication behavior at postintervention and 6-month follow-up. We will also examine the relationship between maternal and infant changes. The study is being conducted in the urban core of a large southern US city, which is one of the fastest growing and most segregated and economically inequitable in the country.

### Clinical Trial Registration and Institutional Review Board Approval

The study is registered as a clinical trial at ClinicalTrials.gov (NCT03464630). Before human subjects’ activity, the full, detailed study protocol #H18217 was approved by the Georgia State University Institutional Review Board (IRB) on November 21, 2017 (see Multimedia Appendix 1 for peer review summary statements of the grant proposal).

### Trial Design, Randomization, and Recruitment

The study uses a two-arm, randomized controlled intent-to-treat trial design, with random assignment in a 1:1 allocation to one of the two parallel mobile intervention conditions. IRB approval is obtained before the involvement of human subjects. Recruitment strategies include the distribution of study information to health and social service agencies serving low-income women. Print materials were provided in agency offices, web-based posts were placed on health and social service websites, and text blasts were sent by community service providers to women on their service lists. Referral is conducted through a project web-based referral system to support mother self-referral, provider referral, and research team referral. Referred mothers are screened by phone to determine their eligibility for inclusion.

### Eligibility Criteria and Participant Characteristics

The eligibility criteria include the following: biological mothers aged 18 years or older with an infant younger than 1 year, who are English speaking, and who meet the Patient Health Questionnaire (PHQ)-2 criteria for elevated depression symptoms [47]. Exclusion criteria at the time of screening include maternal homelessness or shelter residence, major physical or mental illness that would hinder meaningful participation, infant major physical illness, and not having custody of the infant. Inclusion and exclusion criteria are established to ensure that mothers were not burdened by severe stressors that might restrict their ability to participate in the study.

### Sample Size Determination

Sample size determination is based on anticipated effect sizes and the minimum sample size needed to have sufficient power to detect these effects. In this study, a moderate-to-large effect size (Cohen *d*=0.5-1.03) is anticipated for maternal outcomes and a small-to-moderate effect size (Cohen *d*=0.2-0.4) is anticipated for infant outcomes based on the PALS program evidence [18,19], our Baby-Net results [23,46], and evidence of Coping with Depression Course [48] and internet-based cognitive behavioral therapy treatment success [49]. For the smallest anticipated effect sizes (ie, those for infants), to detect these effects within a 2×2 analysis based on α=.05, a sample of n=75 per condition is needed (total n=150). Within this sample size estimation, effects were viewed relative to both high (*r*=0.68) and low (*r*=0.21) repeated measures correlation. We found, with power at 0.95, we could detect an effect as low as Cohen *d*=0.37 (with low repeated correlation) and Cohen *d*=0.23 (with high repeated correlation), and with a power of 0.80, we could detect an effect size as low as Cohen *d*=0.29 (with low repeated correlation) and Cohen *d*=0.18 (with high repeated correlation). To view latent growth curve model maintenance trajectories through follow-up, the number of cases per estimated parameter needs to be sufficient, with a rough guideline of 5:1 [50,51]. Using this guideline, with an estimated sample size of 150, we will have the sample size to estimate 30 parameters, which is sufficient for modeling three time points and a condition predictor.

On the basis of a sample size of 150 needed across the three study assessment points (pre, post, and follow-up), we used anticipated attrition rates to estimate the initial sample size needed to achieve this number. We expect a pre-post attrition rate of 10% and an overall 17% rate at follow-up assessments based on traditional PALS, across a series of randomized control studies, with attrition rates ranging from 9% to 24% [52,53]. In our recent Baby-Net R01 study [45], we observed a 7% attrition from pre to postassessment and 15% at 6-month follow-up working with low-income mothers, some of whom were experiencing elevated levels of depressive symptoms. As such, we estimate that we will need an initial sample of at least 180 mothers to initiate the study, consent, and complete preassessment.

An additional consideration in this study is our need to screen for maternal depression to arrive at an initial sample of 180. The estimated range of depression within our targeted recruitment population of low-income, diverse women ranges from 30% to 50% [11,12]. Hence, at least one out of three women we screen will likely be eligible. This estimate will require us to screen 540 mothers to yield an eligible sample of at least 180 mothers. In previous studies [37,44], 4 out of 5 women who self-select to be screened and are eligible go on to consent to preassessment and intervention. Hence, it is necessary to screen a total sample of 675 to obtain a sample of 180 mothers.

### Intervention and Comparisons

The study includes two parallel mobile internet remote coaching intervention programs that are identical in intervention structure. To reduce literacy demands and maximize accessibility, both programs are video- and narration-based. The structure of intervention delivery for both programs includes the following: (1) web-based administration of a 14-session intervention with video, narration, and activities to present session content and check-in questions to assess knowledge acquisition, recorded in the database for review by both parent and coach; (2) creation of a 5-minute app-collected video of mother-infant interactions for later review by coach and parent; (3) summary of topics; (4) daily activities (homework); (5) participant-rated satisfaction, ease of use, and effectiveness recorded in the database; and (6) weekly video coach calls to coview the mother-infant video. All mothers receive an iPhone with access to their assigned intervention program and unlimited mobile calls, data, and texting. Participants complete the study activities in their homes using these mobile devices.

The content of the MBN intervention sessions on mood improvement focuses on mood monitoring, behavioral activation by increasing mother pleasant activities, and cognitive coping strategies [37]. Parenting content focuses on recognition of infant signals, warm and contingent responding to infant signals, maintaining infant attention and interest, and early language literacy promotion strategies [23,45]. Within the app, mothers receive daily reminders to rate their mood based on their preferred schedule.

As a comparison condition, the Depression and Developmental Awareness (DDAS) program [46] serves as an attention control for the time spent in intervention and remote coaching contact. In contrast to targeting maternal mood and parent practices, the content focuses on awareness of maternal depression and infant developmental milestones.

### Adherence to the Study Protocol and Intervention

Project protocols for consent, assessment, and intervention are used to train all project staff before study initiation. Our consent protocol consists of staff training on a checklist to ensure ethical informed consent. Supervisors observe staff conducting mock consent administration and view their performance relative to the checklist, with a requirement that staff reach 100% accuracy on checklist coverage. For conducting assessments, detailed project protocols are used to train assessors before conducting assessments with mothers. Assessors are trained on assessment protocols focused on providing appropriate assessment instructions, helping mothers understand questions in a manner that will not influence their responses, and administering assessments to mothers verbally if desired by the mother. For interventions, coaches in both conditions are trained to conduct weekly review calls with mothers, rate maternal progress, and complete implementation fidelity checks. A total of 20% of the recorded coach calls are randomly selected for independent completion of fidelity checklists to calculate interobserver agreement of fidelity. In addition, all staff have regular supervision meetings with the principal investigator (PI) to monitor adherence to the consent, assessment, and intervention protocols.

### Study Outcomes

The primary study outcomes include the following: (1) maternal depressive symptoms, (2) parent-sensitive and responsive practices, (3) parent knowledge of infant social-emotional and social-communication behaviors and their promotion, and (4) infant social-emotional and social-communication engagement.

### Data Sources, Collection, and Validity

Following consent, preintervention assessment is completed face-to-face in home or via a mobile video meeting. This comprehensive, multimethod assessment includes interviews, questionnaires, and observational procedures to obtain demographic information and community service receipt and to assess the domains of maternal functioning, including depression symptoms, parenting attitudes and beliefs, parent practices, infant social-emotional and social-communication functioning, and parent-infant interaction. This comprehensive assessment protocol is repeated at postassessment. A 6-month follow-up assessment is administered electronically to assess maternal depression symptoms and parenting knowledge of infant social-emotional and social-communication behaviors and their promotion.

Maternal depressive symptoms and severity are measured using the *PHQ-9*, a 9-item self-report instrument for screening, diagnosing, monitoring, and measuring the severity of depression [54]. Question 9 screens for suicidal ideation. The PHQ-9 has an internal reliability of 0.89 in a primary care setting and 0.86 in an obstetric setting. Maternal parenting behavior, attitudes, beliefs, knowledge, and stress are assessed as follows: the *Landry Parent-Child Interaction Scale observational coding system* [55], designed to assess naturalistic parent-child interaction during play at home, is used to code video-recorded mother-infant interaction behavior. The parent scales of interest in this study relative to maternal responsiveness include ratings of maternal positive affect, warmth, flexibility, and positive verbal content. Relative to maternal negative behavior, scales of interest include ratings of maternal physical intrusiveness as well as verbal and affective negativity. The Landry Parent-Child Interaction Scale has been used in a series of federally funded longitudinal and intervention studies over the past 15 years and has yielded adequate reliability and demonstrated the predictive validity of child social-emotional outcomes [55]. Behaviors will be coded across a semistructured play activity over a 5-minute period. Coders, blinded to intervention conditions and time points, conduct coding based on observing parent-infant interaction videos of participants in both intervention conditions. To assess reliability, 20% of all interactions are scored by 2 independent coders. The *Indicator of Parent Child Interaction-2* [56] includes a brief semistructured book activity, which is video recorded, and will be used to code the following behaviors: (1) mother facilitative behaviors including conveyance of acceptance and warmth, descriptive language, following the child’s lead, and maintaining the child’s interest; (2) mother interrupting behaviors including intrusions, restrictions, and critical comments; (3) infant engagement behaviors, including positive social engagement, follow through, and sustained engagement; and (4) infant behaviors that interfere with engagement, such as fussiness, disruptive behaviors, and withdrawn behavior. The Indicator of Parent-Child Interaction has adequate psychometric features [57] and has been used to assess mother and infant behavior in multiple studies of high-risk infants and in population-based studies of universal interventions to promote early positive parent support behavior [58-60]. To assess reliability, 20% of all interactions are scored by 2 independent coders.

The *Knowledge of Infant Social-Emotional Behavior and Promotion* [45,61] has been used in previous intervention studies and is geared toward an understanding of the concepts of infant social-emotional behavior and its promotion by caregivers, assessing both definitional and applied concept knowledge, with items structured in multiple response formats, including open-ended, true or false, and multiple choice formats. The *Concepts of Development Questionnaire* [62] is a 20-item, Likert-type four-point scale (4=strongly agree and 1=strongly disagree) that assesses parenting beliefs. Specifically of interest in assessing our maternal responsiveness domain, the Concepts of Development Questionnaire focuses on constructs of flexibility and child centeredness, in contrast to parent centeredness. *Parenting Sense of Competence* [63] is a 17-item scale assessing parents’ satisfaction and self-perceived competency in the parenting role. Adequate internal consistency, factor structure, and construct validity have been reported [64]. *The Parenting Stress Index-Short Form* [65] is a 36-item self-report instrument that assesses stress directly associated with the parenting role using a five-point scale to indicate the degree to which that item has been stressful, with validity demonstrated for at-risk mothers [66]. The *Brief Child Abuse Potential (BCAP)* [67] is a 34-item self-report screening instrument that contains seven domains. The primary clinical scale (abuse) comprises six factor scales: distress, rigidity, unhappiness, persecution, loneliness, family conflict, and poverty. In addition, the BCAP contains three validity scales: lie, random response, and inconsistency. Overall, the 24-item BCAP abuse scale has high internal consistency (0.89); temporal stability estimates for the abuse scale are also adequate (ie, 0.91 and 0.75 for 1-day and 3-month intervals, respectively). The *Automatic Thoughts Questionnaire* [68] is a 30-item self-report instrument that measures the frequency of automatic negative thoughts related to depression. It contains four domains: personal maladjustment and desire for change, negative self-concepts and negative expectations, low self-esteem, and helplessness. The Automatic Thoughts Questionnaire has a high internal consistency (0.97). In our own work, we have demonstrated negative thoughts as a mechanistic explanation of Mom-Net intervention effects on maternal depression [69]. The *Revised Dyadic Adjustment Scale* [70] is a 14-item self-report instrument that assesses seven dimensions of relationships in three domains: consensus, satisfaction, and cohesion. Overall, the Revised Dyadic Adjustment Scale has a reliability (Cronbach α) of .90.

Infant social-emotional behavior and development change are assessed as follows: *observed infant behavior in interaction* with the mother was assessed using the Landry Parent-Child Interaction Scale, as described above [55]. Rating scales of interest in assessing infant behaviors include attention or arousal, warmth-seeking, and behavioral regulation. The *Devereux Early Childhood Assessment for Infants* [71] is a 33-item behavior rating scale that assesses child protective factors central to social and emotional health and resilience in infants aged 4 weeks up to 18 months, which displays adequate reliability and validity for use in this study [72].

### Moderating Influences

The *Family Profile Report Form* [73] uses demographic and life course history data, including maternal relationship status and support, health status, psychiatric history, and other services received, to describe study participants and examine the potential moderating effects of the intervention. *Socioeconomic stress* will be assessed using Conger and Elder measure of economic hardship based on their family process model of economic hardship [74], which assesses different areas of financial stress and has been used in many studies to describe important aspects of societal disadvantage in samples [75-77]. *Intervention dosage* will be assessed by electronic recording of the mother: (1) number of sessions and homework completed, (2) time on the web, and (3) number of times the intervention was visited. System activity logs will provide descriptive statistics on (1) time of day at log on; (2) length of time in the intervention; (3) number of times logged on per week; and (4) length of time spent in the information, support, video, and assessment areas of the intervention. Program attrition will be documented, and for these subjects, we will conduct exit interviews to determine the reasons for discontinuation. Table 1 presents our project enrollment, intervention, and assessment schedule.

**Table 1:** SPIRIT (Standard Protocol Items: Recommendations for Interventional Trials) schedule of enrollment, interventions, and assessments for the Mom and Baby Net randomized controlled trial.

Timepoint		Enrollment	Allocation	Postallocation				
		–T1	T0	T1 Preassessment	Intervention (6-8 months)		T2 Postassessment (7-9 months)	T3 Follow-up assessment (13-15 months)
					S1	S4		
**Enrollment**								
	Eligibility screen	✓						
	Informed consent	✓						
	Allocation		✓					
**Interventions**								
	Mom and Baby Net				✓	✓		
	Depression and Developmental Awareness				✓	✓		
**Assessments**								
	Depression screening	✓						
	Demographics			✓				
	Covariates			✓				
	Primary outcomes			✓			✓	✓
	Secondary outcomes			✓			✓	

### Data Management

All data will be deidentified by using a project-specific identification number for each participant. Links between participant names and identification codes will exist in written form only on consent forms, and these forms will be stored in a locked room and file cabinet accessible only to the Georgia State University project staff. In addition, electronic files containing participant identifiers or data will be accessible only to the approved project staff. All nonidentifiable data (ie, those labeled with codes only) will be stored in the same manner in locked file cabinets or on password-protected, secure computer networks.

The data used for progress monitoring will be obtained electronically through the iOS app. State-of-the-art security protocols are used in all data collection and monitoring activities, as used for electronic commerce, using VeriSign SSL. The program app will be established within the Oregon Research Institute network with firewall to maintain security. The program app will be accessible via an iPhone with username and password protection.

### Preliminary Analysis

#### Overview

We will use a systematic approach to construct development to create two parent outcomes (mother positive or negative) and one infant outcome (infant social-emotional functioning). For our outcomes of maternal depression, parenting knowledge, and stress, we will examine single indicants. To view potential intervention moderating effects, we will attempt to create a maternal contextual risk indicator (eg, isolation or support, relationship status, conflict, and economic hardship); for maternal depression before intervention as a potential moderator, we will view depression onset, chronicity, and severity as well as the presence of other treatments, including medication, to examine each of these and their effect on intervention outcomes.

For factor analysis, given appropriate internal consistency and interrater reliability, we will examine questionnaire scales and observational codes using factor analytic techniques [78], retaining a 5:1 subject to parameter ratio. Scale factor loadings above 0.30 and communality estimates above 0.15 will be confirmed within the structural equation modeling methodology to produce fit indices to view how each indicant set fits into their specified domain. If satisfactory, the unit weighting of the standardized score for each indicator will be summed. If not, we will select an index variable within each domain to represent the outcome.

#### Random Assignment and Attention

Although mother-infant pairs will be randomly assigned to the intervention conditions, condition differences may exist due to random sampling failures or differential attrition. To address this issue, a 2×2 (group×attrition status) multivariate analysis of variance (MANOVA) will be performed using the baseline assessment for mother, infant, and contextual risk variables. The presence of a statistically significant group main effect would provide evidence that random assignment was not effective in equating groups. A second possible source of nonequivalence is differential attrition by condition. A significant interaction between group and attrition status provides evidence of differential attrition between groups. In general, analyses will proceed using an intent-to-treat approach, and all participants recruited will be included in subsequent analyses.

#### Missing Data Approach

Multiple imputation will be used to replace missing values following best-practice recommendations [79,80]. Missing data will be imputed using the fully conditional specification, which uses all available data to impute missing data via a sequential regression approach. Missing data points will be replaced with imputed data in 20 data sets, which will be analyzed separately. Model parameters and SEs, which incorporate within and between model variability, will be combined following Rubin methodology [81], as implemented in SPSS version 24 (IBM Corporation) [82].

### Examination of Acute Intervention Effects

Our postassessment n, to examine acute intervention effects, is expected to be 150 (75 per condition). We will initially view intervention effects on our mother or infant outcomes using a 2 (pre-post)×2 (intervention group) repeated measure analysis of variance (ANOVA). We will examine the intercorrelations among outcomes and, if significant, will use a MANOVA approach to examine intervention effects. The *F* test is robust to nonnormality if such nonnormality is caused by skewness rather than outliers. We will take appropriate measures to reduce outlier influences.

To examine the relationship between maternal change (parenting and maternal depression) and infant change, one approach will be to create individual β estimates for mothers and infants using the polynomial contrast function within MANOVA to produce individual trajectory scores reflecting parent and infant change from pre to post that can then be used in external between-condition covariate analysis. The trajectory scores will be subjected to an analysis of covariance, with infant change trajectories as the dependent variable and parent change as the covariate. We will determine whether the parent change covariate is significantly related to the dependent variable (demonstrating that changes in parenting behavior and child functioning covary). We will determine the statistical significance and effect sizes of the intervention group effects (ignoring the covariate). We will then determine if entering the parent functioning covariate modifies the intervention group effect size and statistical significance. If parent change is strongly linked to infant change, then entering the covariate should result in nonsignificant intervention effects. Estimates of covariance-adjusted effect sizes will provide estimates of the proportion of the intervention group effect size, which can be explained by the parenting change variable. We will test for between-group heterogeneity of covariance to determine whether the strength of the association between change in parenting and infant functioning differs by intervention group.

### Examination of Moderating Influences

To evaluate the moderating influences on parent and infant behavior, our first focus is on how maternal depression before intervention (ie, chronicity, severity, and receipt of psychiatric treatment or medication) affects intervention dosage; second, we are interested in the moderating effect of dosage on mother and infant change. For depression experience before intervention, we will examine a 2 (high or low dosage)×2 (intervention condition) ANOVA using maternal depression indicators (eg, chronicity) as the dependent measure. A significant main effect for dosage would indicate that higher levels of maternal depression are found at different dosage levels; it is anticipated that higher depression will be evidenced in the low dosage group. A significant interaction would indicate that a higher level of maternal depression is found within a dosage by condition cell. Although we would not anticipate a significant interaction, we will examine if higher levels of depression are associated with low dosage only within the MBN intervention, which could indicate that the skills focus of MBN learning may have been too intense for highly depressed mothers.

To examine dosage as a moderating influence on mother and infant change, we will use the individual β slope estimates reflecting parent and infant change as the dependent measures in separate 2 (intervention condition)×2 (high or low dosage) ANOVA designs. We hypothesize that a significant interaction term indicating the highest positive change trajectories will be for MBN mothers with high dosage, when compared with low dosage MBN mothers and DDAS mothers, regardless of dosage level. If a significant dose-effect relationship exists within the MBN condition, we will determine if an effective dosage level can be identified that is less than the maximum number of intervention sessions offered.

For contextual risks, analysis will examine Pearson correlations between contextual risk and level of maternal depression and a biserial correlation for the relationship between contextual risk and dosage (high or low) to determine if contextual risks are related to both initial levels of depression and subsequent engagement in intervention. To further examine the contextual risk of mother and infant change, we will form a high or low risk categorical variable based on median split and use the same 2 (intervention condition)×2 (high or low risk). If the main intervention condition effect is significant, we would expect MBN mothers and infants, regardless of risk level, to show the greatest improvements when compared with DDAS mothers. If significant interaction effects occur, we would expect mothers and infants within the MBN condition with lower levels of risk to show the greatest improvement in functioning and, though not statistically significant, that MBN mothers and infants, even in the presence of high risk, would show higher positive change trajectories than those of DDAS mothers and infants.

### Examination of Maintenance of Effects

Maintenance affects will be viewed by a single indicator for maternal depression and parenting knowledge administered at follow-up. To this end, we will examine maternal change trajectories using structural equation modeling methodology, perform latent growth curve model analyses, and include an intervention condition that predicts intercept and slope estimates. This analysis will supplement our aim 2 analyses, and if these follow-up variables generally reflect the acute intervention trajectories, this will provide support for our view of maternal change across time. Given the restricted nature of follow-up assessments, balancing participant burden, and the desire to maximize assessment completion across time, we will view these maintenance trajectories with caution.

### Safety Considerations

Before the study activities, all staff complete human subjects training and participate in safety monitoring and safety responding training under the supervision of the PI and licensed psychologist. Training includes discussion and written responses on study safety monitoring forms, followed by completion of safety monitoring forms to fidelity based on vignette practice. Safety monitoring forms are completed at all assessment time points and upon reporting of any concern about potential harm. Mothers in both conditions receive biweekly automated texts to complete the PHQ-9 [54]. PHQ-9 results are monitored by project coaches for trends in increasing depression severity and harmful thoughts, which trigger an immediate safety response from coaches. All safety monitoring forms that include endorsement of harmful thoughts and required safety response documentation are submitted to the project PI for review. Adverse events and serious adverse events are logged and reported to the IRB and National Institutes of Health (NIH).

## Results

### Overview

The study was funded by the NIH to begin September 1, 2016. Recruitment efforts were initiated immediately following IRB approval on November 17, 2017. A sample of 184 mothers and their infants were recruited into the randomized controlled trial study between February 15, 2018, and March 11, 2021. Study intervention is underway, and we anticipate that follow-up assessments, which mark the end of data collection, will be completed in March 2022. Following the onset of COVID-19, we published a formative descriptive report on recruitment strategies [46] and a descriptive report of progression from referral to intervention initiation for mothers with study experience before the pandemic and mothers with study experience during the pandemic [83].

### Data Availability

Deidentified study data will be made available publicly through a Georgia State University website and through a digital object identifier–linked public repository OpenTrials [84] following a 1-year embargo from the date of publication of primary study outcomes to allow for any commercialization.

## Discussion

This protocol describes a randomized controlled trial to evaluate MBN, a mobile remote coaching intervention to reduce maternal depression, promote sensitive and responsive parenting, and improve infant outcomes during the first postpartum year. We have successfully enrolled a socioeconomically disadvantaged sample of primarily African-American women (N=184) into the trial. We anticipate that, relative to the comparison intervention that is focused on education about maternal depression and infant developmental milestones and matched on technology and coaching structure, women in the MBN intervention will experience greater reductions in depression and gains in sensitive-responsive parenting and that their infants will demonstrate more optimal social-emotional and social-communication behavior.

## Appendix

Multimedia Appendix 1 Peer-review report by the Center for Scientific Review Special Emphasis Panel - Member Conflict: Developmental Risk Prevention, Aging and Social Behavior (National Institutes of Health).

## Abbreviations

ANOVA: analysis of variance
BCAP: Brief Child Abuse Potential
DDAS: Depression and Developmental Awareness
ePALS: e-Play and Learning Strategies
IRB: institutional review board
MANOVA: multivariate analysis of variance
MBN: Mom and Baby Net
NIH: National Institutes of Health
PALS: Play and Learning Strategies
PHQ: Patient Health Questionnaire
PI: principal investigator
