# Supplementary material for: Optimizing Social-Emotional-Communication Development in Infants of Mothers With Depression: Protocol for a Randomized Controlled Trial of a Mobile Intervention Targeting Depression and Responsive Parenting
Source: JMIR Res Protoc. 2021 Aug 18;10(8):e31072. doi: 10.2196/31072 (PMC8411326; doi:10.2196/31072)
Supplement: Multimedia Appendix 1 [file resprot_v10i8e31072_app1.pdf]

SUMMARY STATEMENT

(Privileged Communication)

PROGRAM CONTACT:
LAYLA ESPOSITO
(301) 435-6888
espositl@mail.nih.gov

Release Date: 03/08/2016 04:50 PM
Revised Date:

Application Number: 1 R01 HD086894-01A1

Principal Investigators (Listed Alphabetically):

BAGGETT, KATHLEEN MARIE (Contact)
FEIL, EDWARD GUSTAV

Applicant Organization: UNIVERSITY OF KANSAS LAWRENCE

Review Group: ZRG1 RPHB-W (03)
Center for Scientific Review Special Emphasis Panel
Member Conflict: Developmental Risk Prevention, Aging and Social Behavior

Meeting Date: 02/25/2016
Council: MAY 2016
Requested Start: 09/01/2016
RFA/PA: PA13-302
PCC: CDBB -LE
Dual IC(s): MD, MH, NR

Project Title: Your Strength, Your Baby: Web-Based Remote Coaching to Reduce Maternal Depression and Promote Infant Social-Emotional Health and Development
SRG Action: Impact Score:20 Percentile:5 #
Next Steps: Visit http://grants.nih.gov/grants/next_steps.htm
Human Subjects: 44-Human subjects involved - SRG concerns
Animal Subjects: 10-No live vertebrate animals involved for competing appl.
Gender: 1A-Both genders, scientifically acceptable
Minority: 1A-Minorities and non-minorities, scientifically acceptable
Children: 1A-Both Children and Adults, scientifically acceptable
Clinical Research - not NIH-defined Phase III Trial

Project Year	Direct Costs Requested	Estimated Total Cost
1	499,949	694,239
2	499,966	694,263
3	499,466	693,569
4	499,779	694,003
5	499,975	694,276
TOTAL	2,499,135	3,470,350

ADMINISTRATIVE BUDGET NOTE: The budget shown is the requested budget and has not been adjusted to reflect any recommendations made by reviewers. If an award is planned, the costs will be calculated by Institute grants management staff based on the recommendations outlined below in the COMMITTEE BUDGET RECOMMENDATIONS section.
ADMINISTRATIVE NOTE
BUDGET MODIFICATIONS

1R01HD086894-01A1 Baggett, Kathleen

PROTECTION OF HUMAN SUBJECTS UNACCEPTABLE
COMMITTEE BUDGET RECOMMENDATIONS
ADMINISTRATIVE NOTE

RESUME AND SUMMARY OF DISCUSSION: This application proposes a brief, accessible and integrated parenting intervention (Your Strength Your Baby, YSYB) that combines elements of two separate interventions (Baby-Net and Mom-Net), to target both maternal depression and specific nurturing parent behaviors. The project has the potential to contribute significantly to improving infant social-emotional development. The study addresses an understudied problem and targets vulnerable populations. The design is detailed, thoughtful and feasible, with many innovative methodologies. The investigative team has a history of successful collaboration. This resubmission was responsive to previous critiques. A few concerns were raised during discussion. Given the huge scope of the work, the sample appeared small, the recruitment plan was overly optimistic, the follow up assessment was not well explicated, and further implementation and broader implications were less clearly addressed. However, the panel was confident that success of this much improved and very meritorious study could have a significant impact on the field.

DESCRIPTION (provided by applicant): For infants facing early adversity, intervening early and targeting specific nurturing parent behaviors has proven to be effective in promoting healthy infant social-emotional trajectories. Sadly, maternal depression skyrockets during early infancy, especially for low-income women and interferes with maternal engagement in interventions shown to be effective generally in improving infant social-emotional outcomes. Maternal depression in the first year postpartum constitutes an enormous and costly public health concern with extensive and well-documented detrimental effects on infant parenting and infant life course trajectories. Maternal depression treatments operate in silos, separate from infant parent interventions that target specific parent behaviors shown to promote infant competencies. There is an absence of integrated interventions with demonstrated effectiveness in reducing both maternal depression and promoting infant parenting behavior that builds infant social-emotional competencies. To address the life course needs of depressed mothers and their infants, we need brief, accessible, and integrated interventions that target both maternal depression and specific nurturing parent behaviors shown to improve infant social-emotional trajectories. In our prior programmatic research, we have developed two separate web- based, remote coaching interventions for: (a) parent nurturing behaviors that improve infant outcomes (Baby- Net R34; R01), and (b) maternal depression (Mom-Net R34; R01). Compared to controls, the Baby- Net program demonstrated medium to large effects on observed nurturing parent behavior and on infant social- emotional competencies in the context of play and in the context of book activities. Mom-Net demonstrated low attrition and high levels of feasibility, program use, and satisfaction. Compared to controls, Mom-Net participants demonstrated significant reductions in depression and improved preschool parenting behavior. A substantial advantage of the web-based, remote coaching approach is that it overcomes multiple logistical barriers that often prevent low-income mothers from participating in community/home-visiting treatment programs. While Mom-Net exists for depressed mothers of preschoolers, it is not designed for infant parenting. Moreover, in Baby-Net studies, we found that maternal depression significantly impeded program progress and positive intervention effects. Thus, our prior research on web-based maternal depression and specific nurturing parenting behavior in infancy, provides a strong empirical basis to integrate salient Mom-Net depression content into the Baby-Net program to target depression and specific infant parenting behaviors that promote infant social-emotional competencies and trajectories. We will rigorously test the merged Your Strength-Your Baby (YSYB) intervention effects with 180 low-income mothers with depression and their infants via a 2-arm, intent-to-treat, randomized controlled trial.

PUBLIC HEALTH RELEVANCE: There is a striking absence of accessible, integrated, and evidence-based interventions that target both maternal depression and specific parent behaviors during the first year postpartum shown to improve infant social-emotional trajectories. The proposed work addresses this problem by (a) integrating salient Mom-Net depression content into the Baby-Net program to target depression and support specific infant parenting behaviors related to infant social-emotional competencies and trajectories and (b) rigorously testing the merged Your Strength-Your Baby (YSYB) intervention effects with 180 low-income mothers with depression and their infants via a 2-arm, intent-to-treat, randomized controlled trial. During early infancy, inner-urban, low- income mothers experience depression at a rate of nearly three times the national average. This is a staggering public health concern given that recent research shows that effects are severe for maternal postpartum parenting and infants, with personal and societal costs exceeding those of most medical illnesses and accounting for more disability than any other mental disorder. The proposed innovative work holds enormous scientific and practical impact potential for reducing human and economic costs of depression to mothers, infants, and society.

CRITIQUE 1

Significance: 1
Investigator(s): 2
Innovation: 2
Approach: 2
Environment: 2

Overall Impact: In this resubmitted application, the investigators propose to develop a new web-based intervention, Your Strength Your Baby (YSYB), to improve infant parenting skills in depressed mothers living in low-income housing. YSYB will combine two other web-based programs to optimize an intervention for this population. The Principal Investigator has been responsive to previous review. There is a strong investigative team supporting this application with demonstrated feasibility of working together. Attention to detail is apparent in the assessment battery and data analysis plan. The approach of combining these different successful web-based programs to create this new one (plus additional infant-based expertise) engenders enthusiasm for a product that is very much needed by this under-served population.

1. Significance:

Strengths

- Interventions that increase parenting skills and improve outcomes is a significant goal, especially when the target is women in living in adverse conditions.
- Improving parenting such skills in women with maternal/post-partum depression would have great impact on reducing next generation sequelae of poverty/adversity.

Weaknesses

- None noted.

2. Investigator(s):

Strengths

- Principal Investigator Dr. Baggett is an Adjunct Associate Professor at the University of Kansas Medical Center and the University of Kansas.

- Principal Investigator Dr. Feil is highly qualified to work on the proposed projects on early intervention efforts and has an established relationship with Dr. Baggett (via two publications).
- Dr. Sheeber brings significant intervention expertise by training coaches to provide cognitive behavioral therapy and on designing interventions for depressive individuals (although her expertise is more about adolescents, not moms and infants).
- Drs. Davis, Ammerman, and Landry all bring expertise regarding interventions, working with culturally diverse populations, and web-based programs.
- The inclusion of a bilingual research assistant is a great idea.
- Good attention is paid to moderating details (such as prior or family history, relationship status).

Weaknesses

- The majority of individuals who are consulting will provide 10 hours a year, which is far too little for their proposed level of involvement.
- Dr. Davis describes her role as assisting with making the study culturally-sensitive, whereas her role on the application is described as statistical consultant. Such a discrepancy is disconcerting especially since statistical assessment will be important to determine the success of the project.

3. Innovation:

Strengths

- A web-based intervention for parenting in depressed mothers is mildly innovative.

Weaknesses

- None noted.

4. Approach:

Strengths

- Improving on one web-based program (Baby-Net) by combining what is effective from the other program (Mom-Net) is a solid approach to produce Your Strength Your Baby.
- The use of iPhones for subject renumeration is interesting.
- Strong and appropriate data analytic plan.
- The application is built with an ease-of-use technology to minimize secondary data handling. This is a great practical strength.
- Assessment of outcomes is appropriately attentive to both mother’s and infant’s behavioral changes following intervention.
- The mini-longitudinal assessment of up to 6 months post-intervention will begin to provide insight into lasting effectiveness; this relatively short window is appropriate for the study at this stage.

Weaknesses

- Unclear what happens to the iPhone if the subject drops out of the study – there seems to be no reason to return the phone or how the subject could afford it afterward.

- The lack of independent verification of drug use, such as a tox screen.

5. Environment:

Strengths

- The University of Kansas has established the Juniper Gardens Children's Project, which is further partnered with the Children's Campus of Kansas City, which is a consortium devoted to the support of families and children. This group is well-suited to support the proposed research.
- Fidelity checks on all of the implantation phases is excellent, and appropriately responsive to review.

Weaknesses

- None noted.

Protections for Human Subjects:

Unacceptable Risks and/or Inadequate Protections

- There was no section clearly labeled as "Risk to Subject" or "Adequate Protection from Risk" to be found.
- The Human subjects is not supposed to be a reiteration of the application or an opportunity for more detail, which made it difficult to focus on what is important.
- Potential risk to withdrawal involves using the iPhone. However, if they want to keep the phone this may seem coercive, which would be very much highlighted as they made the call to drop out.

Data and Safety Monitoring Plan (Applicable for Clinical Trials Only):

Acceptable

- A very extensive DSM was included; the minor concern is whether the DSM should also involve a few individuals who are independent of the study.

Inclusion of Women, Minorities and Children:

- Sex/Gender: Distribution not justified scientifically
- Race/Ethnicity: Distribution justified scientifically
- Inclusion/Exclusion of Children under 21: Including ages < 21 justified scientifically
- The Planned Enrollment Chart contains a significant number of males despite the fact that they are not included in the planned study.
- The inclusion of mother-infant dyads for this study scientifically justifies female-only as the sex (although baby may be either male or female) and inclusion of children in this sample, and the demographically-appropriate racial/ethnic inclusion is justified scientifically.

Vertebrate Animals:

Not Applicable (No Vertebrate Animals)

Biohazards:

Not Applicable (No Biohazards)

Resubmission:

- Responsive to previous review.

Resource Sharing Plans:

Unacceptable

- There is no plan stating what is to become of the Your Strength Your Baby web program.

Budget and Period of Support:

Recommended budget modifications or possible overlap identified:

- Personnel are under budget for both time and monies. Consultants are to check a number of details remotely, but are given 10 hours a year (without conference calls in-between).
- The University of Texas' budget does not match the justification.

CRITIQUE 2

Significance: 2
Investigator(s): 2
Innovation: 2
Approach: 3
Environment: 1

Overall Impact: This revised application proposes to develop and study the “Your Strength Your Baby” program (YSYB), a combined parenting and depression treatment intervention. The first year is dedicated to integrating the two currently existing interventions from which YBYS is developed (i.e. PALS and Mom Net); in years 2-5 an RCT will be conducted with 180 postpartum mothers and their infants. The study is well conceptualized and meets an important gap in the literature – the lack of evidence-based interventions that address both maternal depression and concomitant parenting impairments. Moreover, the study addresses barriers to care for mothers who cannot or do not participate in in-person treatment program via its use of an iPhone application to deliver web-based content plus phone coaching. While overall this is a strong application, several small concerns detract from its impact. There is a lack of rationale for, and attention to the measurement timeline (and particularly the three follow-up measurement periods). The use of phone coaching makes it unclear how coach and participant will together watch the weekly videos made by the participant. The contact Principal Investigator’s extensive program of research seems disproportionate to her peer-reviewed publications.

1. Significance:

Strengths

- If successful, the project will address a key barrier to care for mothers with depression and their infants – by providing combined maternal depression treatment and parenting support.
- If successful, the study would advance the field of interventions with mothers suffering from depression by expanding the range of options, particularly for isolated mothers without access to in-person care.

Weaknesses

- None noted.

2. Investigator(s):

Strengths

- Investigators have extensive experience in web-based and home visiting interventions with mothers and children.
- The addition of consultants who are experts in maternal depression and assessment of mother-infant interactions is a plus.
- The Leadership plan is appropriate and the Principal Investigators have worked together extensively over the past 10 years.

Weaknesses

- Principal Investigator Baggett appears to have relatively few publications given the extent of her supported research projects.

3. Innovation:

Strengths

- The combination of maternal depression treatment with parenting skills is innovative.
- Use of video on iPhone is innovative and has potential to advance science by making observational data gathering feasible and much lower-cost

Weaknesses

- Given the extent of the problem (maternal depression and parenting challenges) the inclusion of coaching over the phone vs. web-based coaching is somewhat disappointing. The provision of iPhones offers an opportunity to use applications such as FaceTime to 'live coach' mothers and this is missed with telephone only coaching.

4. Approach:

Strengths

- Well thought-out study, with consideration of many factors related to the intervention (e.g. time to integrate both interventions, and alpha and beta test them).
- Incorporation of several innovative methodological and intervention techniques (e.g. weekly videotaping to watch with coach).

Weaknesses

- Recruitment schedule is very optimistic – it is expected that of the 72 mothers per year (50% of the 144 estimated to be eligible) who return the PHQ-9 cards and complete phone screening, 60 will enroll in the study.
- There is no rationale for using a phone call for coaching given the fact that all participants will have iPhones (and therefore would likely be able to Face Time). It is unclear how participant and therapist will "co-view the parent-child video" on the phone.
- It is described that 6 months is necessary for a 15 session intervention. Is this because parents do not complete the sessions? Will the phone calls be made weekly regardless of whether mothers complete the web components?

- Although the Principal Investigator clarifies in the Introduction to the revised application that "The post-assessment is conducted 6 months after pre-assessment. Follow-up assessments occur at 1- 3, and 6-months after post assessment to assess maintenance of intervention effects" this is still unclear in the narrative. For example, measures simply are labeled pre-, post- or f/u, but it is unclear which of the three f/u points are specified. The Human Subjects section provides a much better clarification of the assessment timeline and which assessments are gathered when – but this information is needed in the Approach section.

5. Environment:

Strengths

- Environment in both Juniper Gardens and at ORI appear to be supportive and able to provide all resources necessary for the successful conduct of the study.

Weaknesses

- None noted.

Protections for Human Subjects:

Acceptable Risks and/or Adequate Protections

- Availability of the Principal Investigator (a licensed psychologist) in case of concerns is important.
- Section on ethical provision of internet interventions is strong.

Data and Safety Monitoring Plan (Applicable for Clinical Trials Only):

Acceptable

Inclusion of Women, Minorities and Children:

- Sex/Gender: Distribution justified scientifically
- Race/Ethnicity: Distribution justified scientifically
- Inclusion/Exclusion of Children under 21: Including ages < 21 justified scientifically

Vertebrate Animals:

Not Applicable (No Vertebrate Animals)

Biohazards:

Not Applicable (No Biohazards)

Budget and Period of Support:

Recommended budget modifications or possible overlap identified:

- There appears to be nothing budgeted for the in-home assessments. An assessment coordinator is budgeted and will conduct the pre-assessments, but there is no mention of her (or anybody else) actually conducting the post and follow-up assessments (each 2hrs) in the homes.

CRITIQUE 3

Significance: 1
Investigator(s): 1
Innovation: 2
Approach: 2
Environment: 1

1. Significance:

Strengths

- The proposed project addresses an important question: can an intervention for depressed mothers of infants be delivered successfully on a smartphone?
- The proposed project aims to combine the goals of intervening in maternal depression and intervening for poor mothers to improve maternal-child interaction.
- A very sizable minority of poor mothers of infants screen positively for depression, which interferes with mother-infant bonding and with infant development.

Weaknesses

- This is a very intensive intervention. Requirements for personnel and equipment are substantial.

2. Investigator(s):

Strengths

- Dr. Baggett and Dr. Feil are both highly experienced in developing, administering and evaluating interventions for parenting and for depression.
- The project team has worked together on interventions for maternal depression, and for maternal-child interaction. This project builds on their experience.
- The project staff is also highly experienced.

Weaknesses

- None noted.

3. Innovation:

Strengths

- This project builds on the success of several similar projects. The innovation is the combination of an intervention for maternal depression and an intervention for maternal-infant bonding.
- The intervention will be delivered remotely, which is designed to make it easier for mothers to participate.
- The control intervention has been carefully designed to mimic the time and attention spent on the tasks without providing parenting advice or treatment for depression. It has proven successful in each of these separately.

Weaknesses

- None noted.

4. Approach:

Strengths

- The remote delivery is a strength of this approach.
- The intervention seems to be engaging and has been well accepted in earlier, similar applications.
- The treatment and the participants are closely monitored by video and in-person visits to assess compliance and outcomes.

Weaknesses

- The application of this intervention on a large scale, the associated costs and challenges, were not addressed.

5. Environment:

Strengths

- Both the University of Kansas and the Oregon Research Institute have excellent facilities for the conduct of this project.

Weaknesses

- None noted.

Protections for Human Subjects:

Acceptable Risks and/or Adequate Protections

Data and Safety Monitoring Plan (Applicable for Clinical Trials Only):

Acceptable

Inclusion of Women, Minorities and Children:

- Sex/Gender: Distribution justified scientifically
- Race/Ethnicity: Distribution justified scientifically
- Inclusion/Exclusion of Children under 21: Including ages < 21 justified scientifically

Vertebrate Animals:

Not Applicable (No Vertebrate Animals)

Biohazards:

Not Applicable (No Biohazards)

Resource Sharing Plans:

Acceptable

Budget and Period of Support:

Recommend as Requested

THE FOLLOWING SECTIONS WERE PREPARED BY THE SCIENTIFIC REVIEW OFFICER TO SUMMARIZE THE OUTCOME OF DISCUSSIONS OF THE REVIEW COMMITTEE, OR REVIEWERS' WRITTEN CRITIQUES, ON THE FOLLOWING ISSUES:

PROTECTION OF HUMAN SUBJECTS (Resume): UNACCEPTABLE

The human subject protection section contains details about the appraoch, which is inappropriate, and makes it difficult to focus on what is important about protection. There were no sections clearly labeled "Risk to Subject" or "Adequate Protection from Risk". There is potential risk to withdrawal that involves using the iPhone.

INCLUSION OF WOMEN PLAN (Resume): ACCEPTABLE

INCLUSION OF MINORITIES PLAN (Resume): ACCEPTABLE

INCLUSION OF CHILDREN PLAN (Resume): ACCEPTABLE

COMMITTEE BUDGET RECOMMENDATIONS:

Consultants are under budget for both time and monies. There appears to be nothing budgeted for conducting the post and follow-up assessments (each 2 hours) in the homes.

ADMINISTRATIVE NOTE:

During the review of this application, reviewers noted that page limits for one or more sections of the application may have been circumvented by including excess text in one or more application sections that do not have specified page limits (e.g., Protection of Human Subjects. In egregious cases, the NIH has the authority to withdraw such applications from review or consideration for funding (NOT-OD-11-021).

Footnotes for 1 R01 HD086894-01A1; PI Name: Baggett, Kathleen Marie

Ad hoc or special section application percentiled against "Total CSR" base.

NIH has modified its policy regarding the receipt of resubmissions (amended applications). See Guide Notice NOT-OD-14-074 at http://grants.nih.gov/grants/guide/notice-files/NOT-OD-14-074.html. The impact/priority score is calculated after discussion of an application by averaging the overall scores (1-9) given by all voting reviewers on the committee and multiplying by 10. The criterion scores are submitted prior to the meeting by the individual reviewers assigned to an application, and are not discussed specifically at the review meeting or calculated into the overall impact score. Some applications also receive a percentile ranking. For details on the review process, see http://grants.nih.gov/grants/peer_review_process.htm#scoring.

MEETING ROSTER

The roster for this review meeting is displayed as an aggregated roster that includes reviewers from multiple CSR Special Emphasis Panels of the Risk, Prevention and Health Behavior Intergrated Review Group
for the 2016/05 council round.
This roster for CSR is available at:
http://public.era.nih.gov/pubroster/Reports?DOCTYPE=SEP&DESFORMAT=PDF&AGENDA_SEQ_NUM_P=303919
